# Dietary intake and risk of metabolic syndrome in long-term survivors of pediatric allogeneic hematopoietic stem cell transplantation

**DOI:** 10.1038/s41409-024-02412-1

**Published:** 2024-09-18

**Authors:** Louise Lindkvist Pedersen, Tina Gerbek, Maria Ebbesen Sørum, Ena Muhic, Terkel Christiansen, Karin Kok, Kaspar Sørensen, Christian Mølgaard, Klaus Müller

**Affiliations:** 1 Department of Pediatrics and Adolescent Medicine, University Hospital Rigshospitalet, Copenhagen, Denmark; 2 Pediatric Nutrition Unit, University Hospital Rigshospitalet, Copenhagen, Denmark; 3 Department of Nutrition, Exercise and Sports, University of Copenhagen, Copenhagen, Denmark; 4 Institute of Clinical Medicine, Faculty of Medicine, University of Copenhagen, Copenhagen, Denmark; 5 Institute for Inflammation Research, Copenhagen University Hospital - Rigshospitalet, Copenhagen, Denmark

**Keywords:** Risk factors, Paediatrics, Epidemiology

## Abstract

We explored the dietary intake and metabolic syndrome (MetS) in 85 survivors of pediatric stem cell transplantation (median age 30 years, median follow-up time 20 years). Overall, the distribution of fatty acid deviated from the recommendations with a higher intake of saturated fat and a lower intake of unsaturated fat but was comparable to that of the background population. The prevalence of MetS was 27%, corresponding to that of the elderly background population. We compared the intake of macronutrients between those with MetS and those without MetS and found that overall fat intake was higher in patients with MetS (36.7E% [range, 27.2–51.2E%] vs. 33,5E% (range, 23.4–45.1E%), *P* = 0.016). Within the subgroup of patients treated with total body irradiation (TBI), we found a higher fat intake in those with MetS (36.8E% (range, 27.2–51.2E%) versus 32.0E% (range, 24.6–42.1E%), *P* = 0.013). This was confirmed in a multivariate analysis adjusted for TBI, sex, and age at follow-up (OR 1.20 (1.06–1.39), *P* = 0.008). Our findings suggest that conditioning with the use of TBI may induce a state of hypersensitivity to the potentially harmful effects of fat in the diet and suggest that this risk of MetS after TBI treatment may be modifiable by dietary changes.

## Introduction

Allogeneic hematopoietic stem cell transplantation (HSCT) is a treatment for children with high-risk hematological cancers and some non-malignant diseases. Increasing survival rates have led to a growing population of long-term survivors and an increased focus on the late effects of the treatment [1]. A prevalent late effect is early onset metabolic syndrome (MetS), a clustering of risk factors associated with type 2 diabetes and cardiovascular disease. While MetS is a condition frequently encountered in the elderly population, 30–50% of young adults treated with HSCT show manifestations of MetS, which underlines the risk of long-term cardio-metabolic disease in these survivors [2–4]. Likewise, in a cross-sectional study of young adult survivors of pediatric HSCT, we found elevated cholesterol, increased insulin resistance, and sarcopenia in 25–50%, and atherosclerotic plaques in 33% of the total cohort, overall comparable with findings seen in individuals above 60 years in the background population [5, 6]. It is well established that inadequate physical activity combined with a diet high in saturated fat and sugar and low in fruit and vegetables increases the risk of MetS [7, 8]. Observational studies have shown that a high intake of saturated and trans fatty acids promotes insulin resistance, whereas monounsaturated, polyunsaturated, and longer-chain n-3 fatty acids appear to improve insulin sensitivity [9]. However, over the past 30 years, legislation in many countries has limited industrially produced trans-fatty acids in foods and is today without any significance for the intake in Denmark [10].

Despite numerous studies that clearly document a significantly higher incidence of MetS in HSCT survivors, there is a gap in our understanding of the impact of lifestyle factors in this group of patients and in particular the role of the diet. Meanwhile, the intensity of the chemotherapeutic regimen and in particular total body irradiation (TBI) are seen as risk factors [11–13]. The aim of this study was to provide a detailed characterization of the diet in long-term survivors of pediatric HSCT and to explore associations between dietary patterns and MetS, thereby paving the way towards evidence-based personalized nutritional interventions to limit cardiovascular disease in these patients.

## Materials and methods

### Study design and population

We conducted a cross-sectional follow-up study in a cohort of adult survivors of pediatric myeloablative allogeneic HSCT in Denmark. Eligible were all patients who underwent HSCT before the age of 18, between January 1980 and January 2018, and who were above 18 years at the inclusion. These patients were part of a previously published study cohort [14].

### Dietary record/Nutrition intake surveys

The participants’ intake of energy and macronutrients was measured in a 3-day dietary record. Participants were instructed to document all food and beverages consumed over two consecutive weekdays and one weekend day, using the web-based software MADLOG [15]. The macronutrient contents for specific brand foods were retrieved from the manufacturers nutritional product labels and for generic foods, the macronutrient contents were retrieved mainly from the national food composition database (Frida) [16]. If any meals of the day were lacking, or if any reported amount seemed unlikely, or if any other uncertainties were present, clarification was obtained in dialogue with the participant.

The dietary distribution of macronutrients was evaluated in energy percentages (E%). To calculate the energy, standard factors of gram to energy were used (fat: 9 kcal (37 kJ); protein: 4 kcal (17 kJ); carbohydrate: 4 kcal (17 kJ)). To evaluate the diets energy distribution in relation to the recommendations the energy percentages (E%) of the macronutritions were calculated.

Results were compared with the Nordic Nutrition Recommendations (NNR 2012) [17] and with previous reports on dietary intake in the background population [18].

### Metabolic syndrome criteria

MetS was defined according to the National Cholesterol Education Program (NCEP) for Adult Treatment Panel III (ATP III) criteria by fulfillment of at least three of the following criteria: fasting plasma glucose (FPG) ≥ 5.6 mmol/L or treatment for diabetes, high-density lipoprotein (HDL) < 1.3 mmol/L in women or <1.03 mmol/L in men, fasting plasma triglyceride (TG) ≥ 1.7 mmol/L or medical treatment of dyslipidemia, abdominal circumference (AC) ≥ 102 cm in men or ≥ 88 cm in women, blood pressure (BP) ≥ 130 mmHg systolic or ≥ 85 mmHg diastolic or medical treatment of hypertension [4].

Patients had been fasting for a minimum of 8 hours before blood tests. Fasting levels of plasma triglycerides, HDL and glucose were analyzed by the Department of Clinical Biochemistry at Copenhagen University Hospital Rigshospitalet in Denmark. Abdominal circumference (cm) was measured as the widest point between the upper iliac crest and lower ribs. Systolic and diastolic BP were calculated as the mean of three measurements taken after at least 15 minutes of rest in a sitting or reclined position.

### Clinical data

Diagnoses and transplantation characteristics were obtained from the medical records. Patients were screened for signs of chronic graft versus host disease (GvHD) according to the National Institutes of Health criteria upon examination [19, 20]. Anthropometric data were measured on site and lists of medications were obtained by interview via patient history and confirmed by the medical records.

### Statistics

Data are shown as median and range unless otherwise indicated. Associations between MetS and binary explanatory variables were evaluated using Fisher’s exact test, and the associations between MetS and continuous explanatory variables were evaluated via logistic regression analyses. Multiple logistic regression models were adjusted for significant covariates but due to collinearity between malignant disease and TBI, only TBI was entered in the multivariate analysis. The same applied to the age at follow-up and the years from transplantation, where only the age at follow-up was entered. The Mann-Whitney U test was used to examine the association between continuous outcomes and binary covariates. In all statistical analyses, a *P* value < 0.05 was considered statistically significant. All statistics were performed using R statistical software version 4.1.0 (R Foundation for Statistical Computing, Vienna, Austria).

## Results

### Patient’s characteristics

**Fig. 1:**
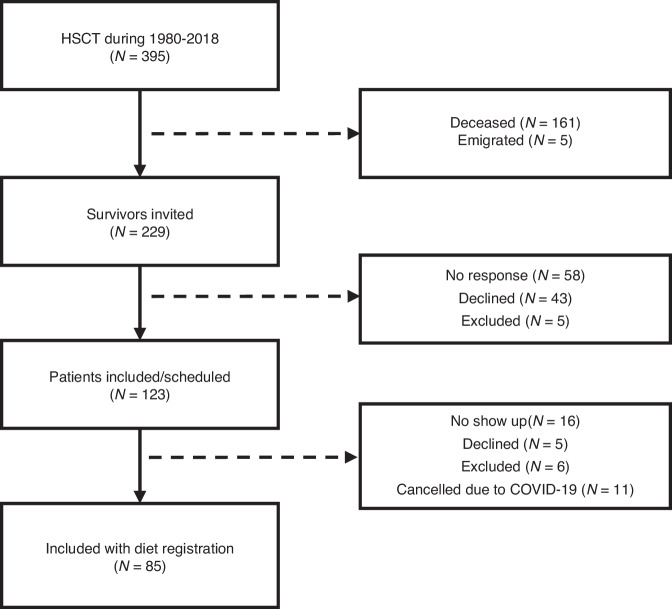
Inclusion flowchart for patients. HSCT Hematopoietic Stem Cell Transplantation.

Of 97 included participants, 85 completed the 3-day dietary registration: 44 males and 41 females. Six were excluded due to a lack of ability to perform the diet registration and 5 declined to participate (Fig. 1). The median age at inclusion was 30.4 years (range: 19.6–53.0) and the median time from transplantation was 19.9 years (range: 5.9–36.9 years). Patient and transplant characteristics are summarized in Table 1.

**Table 1:** Patients and Transplantation characteristics.

Characteristics	Values
Included survivors, *n*	85
No. of males (%)	44 (52)
Patient-related characteristics, median (range)	
Age at follow-up (years)	30.4 (19.6-53.0)
Time from transplantation to examination (years)	19.9 (5.9-36.9)
Age at transplantation (years)	10.9 (0.4-17.9)
Donor age (years)	11.5 (1.7-47.6)
Diagnosis, *n* (%)	
Acute leukaemia	42 (49)
Other malignant disease	15 (18)
Non-malignant disease	28 (33)
Donor type, *n* (%)	
HLA-identical sibling	45 (53)
Matched unrelated donor	23 (27)
Others	17 (20)
Graft type, *n* (%)	
Bone marrow	78 (92)
Peripheral blood	2 (2)
Umbilical cord blood	5 (6)
Conditioning, *n* (%)	
TBI-based conditioning regimes (applied for malign diagnoses)	45 (53)
BU + CY	24 (28)
Other	16 (19)
GvHD, *n* (%)	
Acute GvHD	
Grade 0–I	60 (70)
Grade II–IV	25 (30)
Chronic GvHD	
Yes	21 (25)
No	64 (75)

*HLA* Human leukocyte antigen, *TBI* total body irradiation, *BU* busulfan, *CY* cyclophosphamide; *GvHD* graft versus host disease. Acute GvHD was graded according to the Glucksberg criteria [19, 20].

No significant differences were found between participants and non-participants regarding age at the time of the study, time from HSCT, diagnosis, age at HSCT, conditioning regimen, donor match or development of acute GvHD (data not shown).

### Nutrition characteristics

**Fig. 2:**
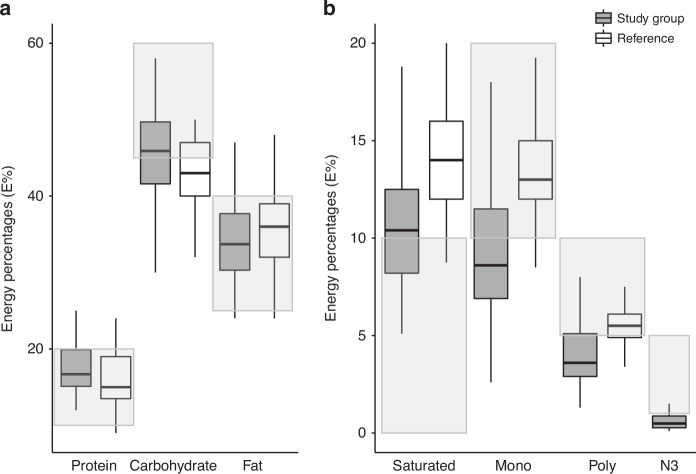
Intake of macronutrients and intake of fatty acid compared with background population and recommendations in E%. **a** Intake of macronutritions, for the included patients (Study group), in energy percentages (E%) compared with the background population (Reference) [18] and the recommendation [17] (shaded area). **b** Intake of fatty acids, for the included patients (Study group), in energy percentages (E%) compared with background population (Reference) [18] and the recommendation (shaded area) [17]. No data is available data on N3 fatty acids in the background population.

The intake of macronutrients was compared with Nordic Nutrition Recommendations 2012 (Fig. 2a, b) [17]. The median intake of carbohydrates was 46E% [range, 24 to 59E%] and in the lower end of the recommended (45–60E%), while median intake of protein and fat were within current recommendations for the background population. Overall, the intake of macronutrients appeared comparable with that reported for the background population (Fig. 2a) [18].

Although the overall fat intake was within the recommendations, the distribution of fatty acids deviated from the recommended with 54% having a higher intake of saturated fat, while 63.5% and 72.9%, respectively, had a lower intake of monounsaturated and polyunsaturated fat, and 81% had a lower intake of N-3 fatty acids. Compared with the background population, there was overall tendency towards a lower intake of both saturated fat and unsaturated fatty acids (Fig. 2b).

A slightly higher intake of protein was associated with non-TBI-based conditioning, transplant for a benign disorder, and absence of acute GvHD during the transplantation (Table 2).

**Table 2:** Associations between dietary components and transplant-related risk factors and GvHD.

Parameter	Carbohydrate		Protein		Fat	
	Percent	*P value*	Percent	*P value*	Percent	*P value*
Treatment groups						
Chemotherapy-based conditioning	45 (25–57)	0.2	17 (11–29)	**0.02**	34 (23–45)	0.9
TBI-based conditioning	46 (23–59)		15 (11–25)		33 (24–51)	
Acute GvHD						
No acute GvHD	44 (25–57)	0.3	18 (11–29)	**0.03**	33 (23–45)	0.7
Acute GvHD	46 (23–59)		16 (11–27)		34 (24–44)	
Chronic GvHD						
No chronic GvHD	45 (23–59)	0.1	16 (11–29)	0.2	34 (23–45)	0.4
Chronic GvHD	47 (28–57)		15 (11–27)		31 (24–51)	
Diagnosis						
Benign disease	44 (30–57)	0.06	18 (12–27)	**0.02**	34 (23–45)	0.6
Malignant disease	47 (23–59)		16 (11–29)		33 (24–51)	
Donor match						
HLA-identical sibling	46 (23–59)	0.6	16 (11–27)	0.8	34 (24–51)	0.6
Other donors	45 (25–57)		16 (13–29)		33 (23–37)	

*TBI* total body irradiation, *GvHD* graft versus host disease, *HLA* Human leukocyte antigen. Statistics: Mann-Whitney U test. Significant *P* values are in bold type. (Median; range).

### Prevalence of MetS

**Fig. 3:**
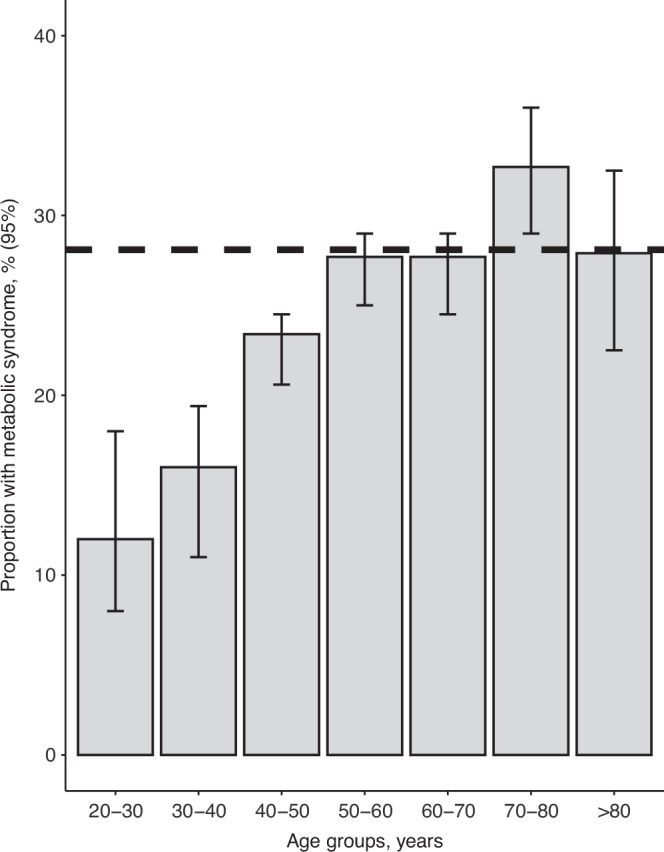
Prevalence of MetS. Prevalence of MetS in the general population by age (data from the Copenhagen General Population Study (*n* = 7981, 4767 males and 3214 females) [21]. The dotted line represents the prevalence of MetS in pediatric HSCT survivors.

The overall prevalence of MetS was 27% (23 of 85) in the HSCT survivors, corresponding to the prevalence of 50- to 80-year-old in the background population (CGPS population) (Fig. 3) [21]. Among patients with MetS, 22/23 patients (95.6%) were treated with TBI (12 GY). Further, male gender, increasing age at follow-up, time from transplant, and malignant disease were all associated with MetS (Table 3). MetS was unrelated to acute GvHD, donor match, and age at the time of transplantation (Table 3).

**Table 3:** Risk factors for MetS after pediatric HSCT.

Factor	Univariate analysis		Multivariate analysis	
	Risk of MetS, OR (95% CI)	*P* value	Risk of MetS, OR (95% CI)	*P* value
Protein (gram)	1.00 (0.98–1.01)	0.96	-	
Protein (E%)	0.88 (0.75–1.02)	0.12	-	*-*
Carbohydrate (gram)	1.00 (0.99–1.01)	0.38	-	
Carbohydrate (E%)	0.97 (0.91–1.04)	0.39	*-*	*-*
Fat (gram)	1.03 (1.00–1.05)	**0.016**	*-*	
Fat (E%)	1.13 (1.03–1.26)	**0.016**	1.20 (1.06–1.39)	**0.008**
Saturated fat (gram)	1.05 (1.00–1.09)	**0.047**	-	
Saturated fat (E%)	1.14 (0.98–1.34)	0.08	-	
Monounsaturated fat (gram)	1.04 (0.99–1.09)	0.12	-	
Monounsaturated fat (E%)	1.05 (0.92–1.19)	0.43	-	
Polyunsaturated fat (gram)	1.04 (0.92–1.19)	0.51	-	
Polyunsaturated fat (E%)	0.94 (0.70–1.23)	0.68	-	
Sex (male)	3.32 (1.22–10.74)	**0.015**	1.73 (1.01–1.20)	**0.02**
Age at follow up (years)	1.12 (1.06–1.20)	**0.0002**	1.09 (1.01–1.20)	**0.04**
Age at transplantation (years)	1.09 (0.09–1.22)	0.14	-	-
Time from transplantation (years)	1.11 (1.04–1.19)	0.0015	-	
Donor age	1.03 (0.99–1.07)	0.19	-	-
Treatment groups				
Chemotherapy-based conditioning	Reference	-		
TBI-based conditioning	44.3 (6.8–961.3)	**<0.0001**	32.42 (4.87–665.1)	**0.002**
Acute GvHD				
No acute GvHD	Reference	-	-	-
Acute GvHD	2.1 (0.7–6.8)	0.2	-	-
Chronic GvHD				
No chronic GvHD	Reference	-	-	-
Chronic GvHD	0.8 (0.2–2.6)	0.8	-	-
Diagnosis				
Nonmalignant disease	Reference	-	-	-
Malignant disease	16.6 (2.6–357.8)	**0.0005**	**-**	**-**
Donor match-				
HLA-identical sibling	Reference	-	-	-
Other donors	1.5 (0.6–4.3)	0.5	-	-

*OR* odds ratio, *CI* confidence interval, *E%* energy percentage, *TBI* total body irradiation, *GvHD* graft versus host disease, *HLA* Human leukocyte antigen.

Statistics: Univariate analysis: Simple logistic regression and Fisher’s exact test. Multivariate analysis: logistic model. Significant *P* values are in bold type.

### Prevalence of MetS and nutritional intake

**Fig. 4:**
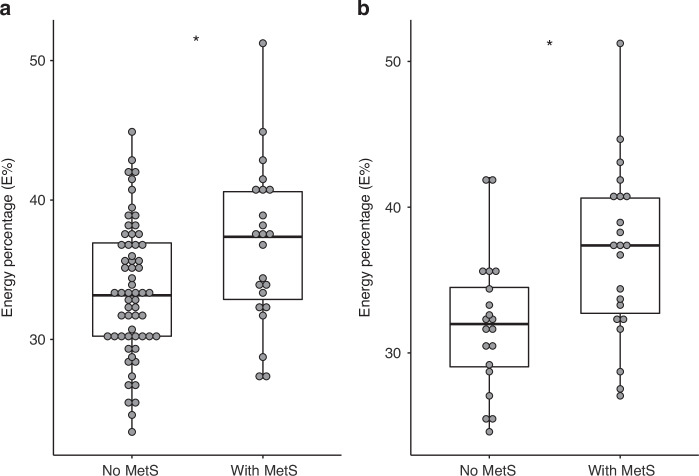
Association between MetS and fat intake in E%. **a** All included patients by MetS status. Patients with MetS had a higher intake of fat in E% (*P = .016). **b** Showing only patients conditioned with TBI by MetS status. Patient with MetS had a higher intake of fat (*P = .013). Simple logistic regression; see Table 2.

We compared the intake of macronutritions between those who met the criteria for MetS and those without MetS and found that the overall fat intake in E% was higher in patients with MetS than in patients without MetS (36.7E% [range, 27.2 to 51.2E%] versus 33,5E% [range, 23.4 to 45.1E%] (*P* = 0.016)) (Fig. 4a), (Table 2).

As above stated, TBI was associated with an increased risk of MetS, but in a stratified analysis of patients treated with TBI (*n* = 42), we found that the risk of MetS was restricted to those patients having a higher fat intake (36.8E% [range: 27.2–51.2E%] versus 32.0E% [range: 24.6–42.1E%], (*P* = 0.013)) (Fig. 4b). In a multivariate analysis adjusted for TBI, sex and age at follow-up, fat intake (E%) remained and independent risk-factor of MetS (Table 2). Of note, the levels of fat intake in the TBI-treated group with MetS were close to the recommendations and comparable with the intake in the general population.

Finally, we examined associations between each individual MetS criteria and fat intake. We found that increased abdominal circumference (*P* = 0.02), increased fasting plasma glucose (*P* = 0.02) and decreased HDL cholesterol (*P* = 0.02) were associated with higher intake of total dietary fat. The remaining criteria showed no significant associations.

## Discussion

An increased risk of MetS is a well-recognized late effect after HSCT in childhood [22–24] and has been associated with the intensity of the conditioning regimen, but no previous study has investigated the potential additive role of the diet in the development of MetS in these patients. We here confirm an increased prevalence of MetS among young adults at a frequency comparable with that seen in the elderly background population. Despite an overall fat intake similar to that reported for the background population, we observed that MetS in TBI-treated patients was associated with a significantly higher fat intake than seen in TBI-treated patients without MetS, highlighting the importance of a reduced fat intake in HSCT-survivors previously treated with TBI. Furthermore, we extend these studies by finding that an increased content of fat in the diet is associated with an increased risk for the development of MetS in patients treated with TBI.

Although these patients appeared to have a diet overall comparable with the background population the dietary patterns in our patients were deviating from the recommendations, as the distribution of the fatty acid indicated a higher intake of saturated fatty acids and a lower intake of both mono- and polyunsaturated fatty acids. This dietary pattern is associated with increased risk of MetS in the general population (age 45–64) both male and female [25].

Poor eating habits may appear early after cancer treatment. A study of dietary intake in young childhood cancer survivors under the age of 13 years, studied less than 5 years after treatment, showed an excessive energy intake [26]. In addition, long-term cancer survivors reported personal barriers for restricting high-fat food intake [27].

The reason for this dietary pattern is unclear. However, the early period after HSCT with severe side effects such as mucositis, nausea, vomiting, and diarrhea, often leads to insufficient oral intake and reduced gastrointestinal absorption. Accordingly, patients may be encouraged to maintain enteral intake in any form possible, potentially resulting in an insufficient and unbalanced diet, which may continue for a longer period, carrying the risk of an unhealthy lifestyle at longer term.

The recognition of the gut microbiome’s role in MetS has been growing due to emerging evidence suggesting its influence on various metabolic processes [28]. Studies addressing changes in gut microbiota during chemotherapy for childhood acute lymphatic leukemia (ALL) have identified antibiotics, immunosuppression, dietary changes, and direct toxicity as key factors contributing to alterations in the intestinal ecosystem [29, 30]. As the use of antibiotics has changed over time, the disturbance of the gut microbiome and the related metabolic effects may have changed in parallel, impacting the risk of MetS. Although we attempted to address this in our statistical analyses of MetS risk factors by including follow-up time, a strong correlation between age and time of follow-up hampered definite conclusions. However, understanding and manipulating the microbiome through diet, lifestyle changes, and targeted use of antibiotics hold significant potential for preventing and managing MetS.

The findings in the present study may be of importance for a more detailed understanding of potential strategies to reduce the high frequency of MetS following HSCT, since dietary habits are potentially modifiable, in contrast to the central elements in the treatment. Indeed, the use of TBI appears to remain a mainstay in the conditioning in patients with ALL, in the near future at least, since a recent pediatric clinical trial proved the superiority of this treatment in terms of overall survival in comparison with non-TBI regimens in patients with ALL [31]. The families and children may, however, be unaware of their risk of chronic disease, lessening the motivation to change their lifestyle [32], and also diet and late events may appear of lower priority in these families, hidden by the trauma of the previous potentially fatal disease. To prevent the onset of chronic disease such as MetS, through lifestyle changes it is necessary to recognize the complexity of the problem. Eating habits are not controlled in isolation by a single factor, but are formed in the stream of events, actions, and relationships, characterized by economic, social cultural, and psychological factors [33]. A health-promoting effort in relation to HSCT patients’ dietary habits will advantageously be based on a broad understanding of the treatment’s impact on children and their families alongside other lifestyle factors, such as physical activity and smoking.

The strengths of this study include the relatively long follow-up period (median 20 years) in a population-based study, representing the breadth of HSCT. Further, the dietary records are self-reported through MADLOG, allowing easy registration in real-time, compared to e.g., the traditional 24-hours recall interview or food frequency questionnaires that include recall bias [34].

While our findings are important given the lack of studies in this area, this study does have some limitations. Due to overlaps in the potential clinical risk factors of MetS, in particular the use of TBI and malignant disease, we were not able to evaluate these combined risk factors selectively. This limitation is potential important because several factors related to the diagnoses and the primary treatment before referral to HSCT may have increased the risk of developing MetS. In particular, we cannot exclude that the use of steroids during the induction phase of ALL treatment could lead to durable changes in eating habits that could predispose to development of the metabolic syndrome. A conclusive evaluation of this is, however, hampered by the fact that several factors in ALL-baseline treatment have changed over time including the composition of the cytotoxic treatment in addition to changes in types of steroids, dosage, and treatment schedules.

As to the dietary patterns, these were self-reported (via MADLOG) rather than directly measured, and subjects may have under-reported caloric intake. Misreporting, and especially under-reporting in dietary surveys is a general problem. Having to register one’s diet for a period, may consciously or unconsciously, lead to dietary changes during the registration period [18, 34]. Indeed, a tendency for selective underreporting has been observed in previous studies, as especially the unhealthy components such as fat and sugar seem underreported, while healthy items such as e.g., vegetables appear to be overreported [35]. Accordingly, the findings in the present study of suboptimal dietary fat-profile are most certainly an underestimation.

To achieve more precise information on the role of the diet in the development of cardiometabolic late effects prospective intervention trials are needed to establish evidence- and risk-based dietary guidelines for preventing cardiovascular disease and other treatment-related late effects among adult survivors of HSCT. Furthermore, the cross-sectional design does not allow for final conclusions regarding casualty, a weakness shared by the previous studies of MetS in HSCT survivors, as well as similar studies in the background population.

In conclusion, long-term survivors of HSCT tend to have a deviating dietary distribution of fatty acids compared with the recommended, although largely comparable with that of the background population. The study confirms that TBI is a main risk factor for MetS post-HSCT, but within this group of heavily treated patients, fat intake remains an independent risk factor. This is of clinical importance because it underlines the importance of an individualized precision diet to reduce the risk of cardio-metabolic disease in survivors of HSCT with a specific focus on patients treated with TBI.

## Data Availability

The data that support the findings of this study are available from the corresponding author upon reasonable request.

## References

[1] Miano M, Labopin M, Hartmann O, Angelucci E, Cornish J, Gluckman E, et al. Haematopoietic stem cell transplantation trends in children over the last three decades: a survey by the paediatric diseases working party of the European Group for Blood and Marrow Transplantation. Bone Marrow Transpl. 2007;39:89–99. 10.1038/sj.bmt.1705550 17213848

[2] Sundholm JKM, Suominen A, Sarkola T, Jahnukainen K. Early arterial intimal thickening and plaque is related with treatment regime and cardiovascular disease risk factors in young adults following childhood hematopoietic stem cell transplantation. J Clin Med. 2020;9:1–13. 10.3390/jcm9072208 PMC7408962 32668566

[3] Nottage KA, Ness KK, Li C, Srivastava D, Robison LL, Hudson MM. Metabolic syndrome and cardiovascular risk among long-term survivors of acute lymphoblastic leukaemia - From the St. Jude Lifetime Cohort. Br J Haematol. 2014;165:364–74. 24467690 10.1111/bjh.12754 PMC4271734

[4] Grundy SM, Cleeman JI, Daniels SR, Donato KA, Eckel RH, Franklin BA, et al. Diagnosis and management of the metabolic syndrome: An American Heart Association/National Heart, Lung, and Blood Institute scientific statement. Circulation. 2005;112:2735–52. 16157765 10.1161/CIRCULATIONAHA.105.169404

[5] Mejdahl Nielsen M, Mathiesen S, Suominen A, Sørensen K, Ifversen M, Mølgaard C, et al. Altered body composition in male long-term survivors of paediatric allogeneic haematopoietic stem cell transplantation: impact of conditioning regimen, chronic graft-versus-host disease and hypogonadism. Bone Marrow Transpl. 2021;56:457–60. 10.1038/s41409-020-01038-3 32879430

[6] Mathiesen S, Uhlving HH, Buchvald F, Hanel B, Nielsen KG, Müller K. Aerobic exercise capacity at long-term follow-up after paediatric allogeneic haematopoietic SCT. Bone Marrow Transpl. 2014;49:1393–9. 10.1038/bmt.2014.172 25111515

[7] Tian Y, Su L, Wang J, Duan X, Jiang X. Fruit and vegetable consumption and risk of the metabolic syndrome: A meta-analysis. Public Health Nutr. 2018;21:756–65. 29151369 10.1017/S136898001700310X PMC10260986

[8] Hoyas I, Leon-Sanz M. Nutritional challenges in metabolic syndrome. J Clin Med. 2019;8:1–11. 10.3390/jcm8091301 PMC6780536 31450565

[9] Pereira MA, Kottke TE, Jordan C, O’connor PJ, Pronk NP, Carreón R. Preventing and managing cardiometabolic risk: the logic for intervention. Int J Environ Res Public Health. 2009;6:2568–84. 20054455 10.3390/ijerph6102568 PMC2790093

[10] Stender S, Dyerberg J. Influence of trans fatty acids on health. Ann Nutr Metab. 2004;48:61–66. 14679314 10.1159/000075591

[11] Bielorai B, Pinhas-Hamiel O. Type 2 Diabetes Mellitus, the metabolic syndrome, and its components in adult survivors of acute lymphoblastic leukemia and hematopoietic stem cell transplantations. Curr Diab Rep*.* 2018; 18. 10.1007/s11892-018-0998-0 29671081

[12] Paris C, Yates L, Lama P, Zepeda AJ, Gutiérrez D, Palma J. Evaluation of metabolic syndrome after hematopoietic stem cell transplantation in children and adolescents. Pediatr Blood Cancer. 2012;59:306–10. 22302361 10.1002/pbc.24104

[13] Oudin C, Simeoni M-C, Sirvent N, Contet A, Begu-Le Coroller A, Bordigoni P, et al. Prevalence and risk factors of the metabolic syndrome in adult survivors of childhood leukemia. Blood. 2011;17:4442–8. 10.1182/blood-2010-09-304899 21278355

[14] Gerbek T, Thomsen BL, Muhic E, Christiansen T, Sørensen K, Ifversen M, et al. Metabolic syndrome as a late effect of childhood hematopoietic stem cell transplantation – A thorough statistical evaluation of putative risk factors. Pediatr Transplant. 2023;27:e14530. 10.1111/petr.14530 37069730

[15] MADLOG. https://www.madlog.dk/ (accessed 18 Jan 2022).

[16] Frida. https://frida.fooddata.dk/ (accessed 16 Mar 2022).

[17] Sandström B, Lyhne N, Pedersen JI, Aro A, Thorsdottir I, Becker W. *Nordic nutrition: Recommendations 2012*. Copenhagen: Nordic Council of Ministers; 2012.

[18] Pedersen AN, Christensen T, Matthiessen J, Knudsen VK, Rosenlund-Sørensen M, Biltoft-Jensen A, et al. *Dietary habits in Denmark 2011–2013*. Copenhagen: Danish Ministry of Food, Agriculture and Fisheries; 2015.

[19] Jagasia MH, Greinix HT, Arora M, Williams KM, Wolff D, Cowen EW, et al. National Institutes of Health Consensus Development Project on Criteria for Clinical Trials in Chronic Graft-versus-Host Disease: I. The 2014 Diagnosis and Staging Working Group Report. Biol Blood Marrow Transplant. 2015;21:389–401.e1. 25529383 10.1016/j.bbmt.2014.12.001 PMC4329079

[20] Glucksberg H, Storb R, Fefer A, Buckner CD, Neiman PE, Clift RA, et al. Clinical manifestations of graft-versus-host disease in human recipients of marrow from HL-A-matched sibling donors. Transplantation. 1974;18:295–304. 4153799 10.1097/00007890-197410000-00001

[21] W Marott SC, Nordestgaard BG, Tybjaerg-Hansen A, Benn M. Components of the metabolic syndrome and risk of type 2 diabetes. J Clin Endocrinol Metab. 2016;101:3212–21. 27285293 10.1210/jc.2015-3777

[22] Baker KS, Chow E, Steinberger J. Metabolic syndrome and cardiovascular risk in survivors after hematopoietic cell transplantation. Bone Marrow Transpl. 2012;47:619–25. 10.1038/bmt.2011.118 PMC13441451 21643022

[23] Muhic E, Mathiesen S, Nielsen MM, Suominen A, Sørensen K, Ifversen M, et al. Metabolic syndrome in male survivors of pediatric allogeneic hematopoietic stem cell transplantation: impact of total body irradiation, low-grade inflammation, and hypogonadism. Transpl Cell Ther. 2021;27:1–8. 10.1016/j.jtct.2021.05.025 34091072

[24] Han TS, Gleeson HK. Long-term and late treatment consequences: Endocrine and metabolic effects. Curr Opin Support Palliat Care. 2017;11:205–13. 28661901 10.1097/SPC.0000000000000289

[25] Lutsey PL, Steffen LM, Stevens J. Dietary intake and the development of the metabolic syndrome. The Atherosclerosis Risk in Communities Study. Am J Clin Nutr. 2008;88:1395–402. 10.1161/CIRCULATIONAHA.107.716159 18212291

[26] Cohen J, Wakefield CE, Fleming CAK, Gawthorne R, Tapsell LC, Cohn RJ. Dietary intake after treatment in child cancer survivors. Pediatr Blood Cancer. 2012;58:752–7. 21850679 10.1002/pbc.23280

[27] Arroyave WD, Clipp EC, Miller PE, Jones LW, Ward DS, Bonner MJ, et al. Childhood cancer survivors’ perceived barriers to improving exercise and dietary behaviors. Oncol Nurs Forum. 2008;35:121–30. 18192161 10.1188/08.ONF.121-130

[28] Velasquez MT. Altered gut microbiota: A link between diet and the metabolic syndrome. Metab Syndr Relat Disord. 2018;16:321–8. 29957105 10.1089/met.2017.0163

[29] Wen Y, Jin R, Chen H. Interactions between gut microbiota and acute childhood leukemia. Front Microbiol. 2019;10:1300. 10.3389/fmicb.2019.01300 PMC6593047 31275258

[30] Rotz SJ, Dandoy CE. The microbiome in pediatric oncology. Cancer. 2020;126:3629–37. 32533793 10.1002/cncr.33030 PMC7678566

[31] Peters C, Dalle J, Locatelli F, Poetschger U, Sedlacek P. Total body irradiation or chemotherapy conditioning in childhood ALL: A Multinational, Randomized, Noninferiority Phase III Study. J Clin Oncol. 2021;39:295–307. 10.1200/JCO.20.02529 PMC8078415 33332189

[32] Nathan PC, Ford JS, Henderson TO, Hudson MM, Emmons KM, Casillas JN, et al. Health behaviors, medical care, and interventions to promote healthy living in the Childhood Cancer Survivor Study cohort. J Clin Oncol. 2009;27:2363–73. 19255308 10.1200/JCO.2008.21.1441 PMC2738646

[33] Hardcastle SJ, Thøgersen-Ntoumani C, Chatzisarantis NLD. Food choice and nutrition: a social psychological perspective. Nutrients. 2015;7:8712–5. 26665419 10.3390/nu7105424 PMC4632444

[34] Biltoft-Jensen A, Matthiessen J, Rasmussen LB, Fagt S, Groth MV, Hels O. Validation of the Danish 7-day pre-coded food diary among adults: Energy intake v. energy expenditure and recording length. Br J Nutr. 2009;102:1838–46. 19650967 10.1017/S0007114509991292

[35] Heitmann BL, Lissner L, Osler M. Do we eat less fat, or just report so? Int J Obes. 2000;24:435–42. 10.1038/sj.ijo.0801176 10805500

